# Three Constituents of *Moringa oleifera* Seeds Regulate Expression of Th17-Relevant Cytokines and Ameliorate TPA-Induced Psoriasis-Like Skin Lesions in Mice

**DOI:** 10.3390/molecules23123256

**Published:** 2018-12-10

**Authors:** Nuan Ma, Qin Tang, Wan-Ting Wu, Xin-An Huang, Qin Xu, Guang-Li Rong, Song Chen, Jian-Ping Song

**Affiliations:** 1 Institute of Tropical Medicine, Guangzhou University of Chinese Medicine, Guangzhou 510405, China; 13710372234@163.com (N.M.); todayfly2003@163.com (Q.T.); wuwanting73@163.com (W.-T.W.); xahuang@chinmednetworks.org (X.-A.H.); xuqin@gzucm.edu.cn (Q.X.); guanglirong2018@126.com (G.-L.R.); 2 Sci-tech Industrial Park, Guangzhou University of Chinese Medicine, Guangzhou 510445, China

**Keywords:** *Moringa oleifera*, niazirin, marumoside A, sitosterol-*3*-*O*-*β*-d-glucopyranoside, Th17, psoriasis

## Abstract

As a folk medicine, *Moringa oleifera* L. is used effectively to treat inflammatory conditions and skin diseases. However, its mechanism of action is not well understood, limiting its medical use. We isolated and identified three compounds, namely niazirin, marumoside A and sitosterol-*3*-*O*-*β*-d-glucoside, from the seeds of *Moringa oleifera*, and studied their effects on the expression of Th17-relevant cytokines (IL-12/IL-23 p40, IL-17A, IL-22 and IL-23 p19) using lipopolysaccharide-stimulated THP-1 cells. Additionally, as Th17 plays a critical role in the pathogenesis of psoriasis, we used a *12*-*O*-tetradecanoylphorbol-*13*-acetate (TPA)-induced psoriasis-like skin lesion mouse model to study their potential therapeutic application in vivo. The compounds suppressed the expression of IL-12/IL-23 p40, IL-17A, IL-22 and IL-23 p19 in vitro, and in vivo they ameliorated psoriasis-like skin lesions, decreased IL-17A mRNA expression, and increased the expression of keratinocyte differentiation markers. To our knowledge, this is the first report regarding the mechanism and therapeutic application of *Moringa oleifera* seeds to treat psoriasis-like lesions in vivo.

## 1. Introduction

*Moringa oleifera* L. (*M*. *oleifera*), also known as horseradish tree, drumstick tree, benzolive tree or ben oil tree, is widely cultivated in Africa, tropical Asia, Latin America and the Pacific Islands [1]. The plant is well known for its industrial and traditional medicine uses, including antibiosis or malaria treatment and treatment of typhoid fever, parasitic diseases, genitourinary ailments, hypertension, inflammatory diseases, swellings, skin diseases, hypoglycemia and diabetes [1,2,3,4,5,6]. In recent years, many studies have reported pharmacological properties of *M. oleifera* in vitro and in vivo, including antihypertensive, hepatoprotective, diuretic, cholesterol lowering, anti-inflammatory, antibacterial and antitumor activities [5,7,8,9]. Several studies have reported that the ethyl acetate fraction of *M. oleifera* significantly inhibited both lipopolysaccharide (LPS)-induced production of nitric oxide and pro-inflammatory cytokines in macrophages in a concentration-dependent manner, and the TNF-α, IL-6 and IL-8 production induced by a cigarette smoke extract in human macrophages [10,11]. Nevertheless, the bioactive constituents and their anti-inflammatory mechanisms have not been well elucidated, restricting their potential clinical application.

THP-1 is a human leukemia monocytic cell line, which has been used extensively to study monocyte/macrophage functions, mechanisms and signaling pathways [12], as well as the inflammation-modulating effects of food-derived compounds [13]. In this study, we isolated constituents of *M. oleifera* seeds and studied their effects on the expression of Th17-relevant cytokines using LPS-stimulated THP-1 cells. Considering that Th17-relevant cytokines are critically involved in the pathogenesis of psoriasis [14], we further evaluated their potential therapeutic application in psoriasis using a *12*-*O*-tetradecanoylphorbol-*13*-acetate (TPA)-treated mouse model.

## 2. Results

### 2.1. C.haracterization of Isolated Compounds

**Figure 1:**
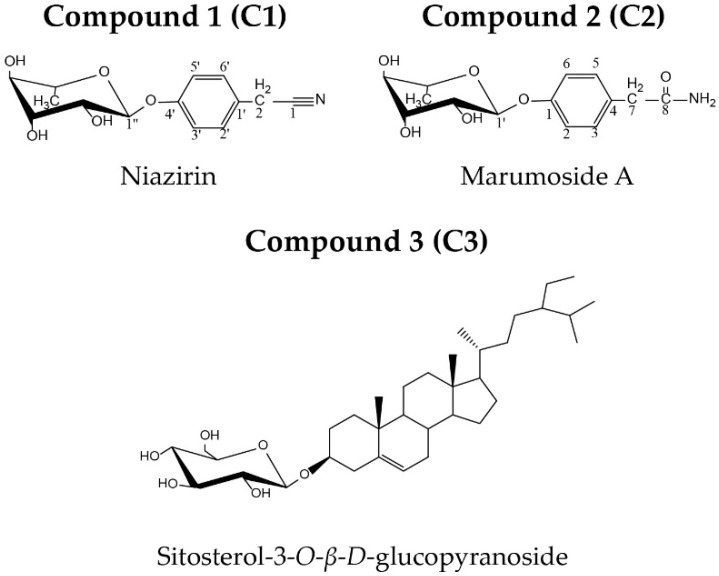
Structures of compounds **1**, **2** and **3**.

Three constituents were purified from *M. oleifera* with purities greater than 90% determined by high performance liquid chromatography (HPLC). Their structures are shown in Figure 1, and their spectral data are listed below:

Niazirin (C1): white needles (from MeOH), positive high-resolution electrospray ionisation mass spectrometry (HRESIMS) *m*/*z*: 302.0989 [M + Na]^+^ (calcd. for C_14_H_17_NO_5_Na, 302.0999). ^1^H-NMR (nuclear magnetic resonance) spectrum (400 MHz, CD_3_OD) *δ* 7.28 (2H, d, *J* = 8.0 Hz, H-3 and 5), 7.07 (2H, d, *J* = 8.0 Hz, H-2 and 6), 5.44 (1H, s, H-1′), 4.00 (1H, br, H-2′), 3.84 (1H, m, H-3′), 3.83 (2H, s, H-7), 3.62 (1H, m, H-5′), 3.46 (1H, t, *J* = 9.5 Hz, H-4′), 1.22 (3H, d, *J* = 6.2 Hz, H-6′). ^13^C-NMR (100 MHz, CD_3_OD) *δ* 157.5 (C-1), 118.2 (C-2 and 6), 130.5 (C-3 and 5), 125.9 (C-4), 22.9 (C-7), 120.0 (C-8), 100.0 (C-1′), 72.1 (C-2′), 72.3 (C-3′), 73.9 (C-4′), 70.8 (C-5′), 18.2 (C-6′). The spectral data were in accordance with those reported [15].

Marumoside A (C2): white needles (from MeOH), positive HRESIMS *m*/*z*: 320.1106 [M + Na]^+^ (calcd. for C_14_H_19_NO_6_Na, 320.1105). ^1^H-NMR (400 MHz, CD_3_OD) *δ* 7.24 (2H, d, *J* = 8.4 Hz, H-2′ and 6′), 7.02 (2H, d, *J* = 8.4 Hz, H-3′ and 5′), 5.42 (1H, s, H-1″), 4.02 (1H, m, H-2″), 3.87 (1H, dd, *J* = 9.6, 3.3 Hz, H-3″), 3.65 (1H, m, H-5″), 3.48 (1H, t, *J* = 9.6 Hz, H-4″), 3.47 (2H, s, H-2), 1.23 (3H, d, *J* = 6.2 Hz, H_-_6″). ^13^C-NMR (100 MHz, CD_3_OD) *δ* 177.4 (C-1), 42.7 (C-2), 130.7 (C-1′), 131.4 (C-2′, C-6′), 117.8 (C-3′, C-5′), 156.9 (C-4′), 100.0 (C-1″), 72.2 (C-2″), 72.3 (C-3″), 73.9 (C-4″), 70.7 (C-5″), 18.2 (C-6″). The data was consistent with those reported [16].

Sitosterol-*3*-*O*-*β*-d-glucopyranoside (C3): white powder (from pyridine), ^1^H-NMR (400 MHz, pyridine-*d*_5_) *δ* 5.29 (1H, m, H-6), 5.07 (1H, d, H-1′), 4.47 (1H, dd, *J* = 11.5 Hz, H-6′), 4.29 (2H, m, *J* = 7.7 Hz, H-3′ and H-4′), 3.95 (1H, br, *J* = 8.2 Hz, H-2′), 3.04 (1H, m, H-5′), 1.00 (3H, d, *J* = 6.5 Hz, H-21), 0.95 (3H, s, H-19), 0.94 (3H, d, *J* = 6.8 Hz, H-26), 0.87 (3H, t, H-29), 0.86 (3H, d, *J* = 7.5 Hz, H-27), 0.64 (3H, s, H-18). ^13^C-NMR (100 MHz, pyridine-*d*5) *δ* 38.1 (C-1), 30.6 (C-2), 79.1 (C-3), 39.5 (C-4), 141.6 (C-5), 122.5 (C-6), 32.4 (C-7), 32.3 (C-8), 50.6 (C-9), 37.5 (C-10), 21.9 (C-11), 39.6 (C-12), 43.1 (C-13), 57.4 (C-14), 25.1 (C-15), 29.1 (C-16), 56.9 (C-17), 12.6 (C-18), 19.7 (C-19), 37.0 (C-20), 19.6 (C-21), 34.5 (C-22), 26.7 (C-23), 46.7 (C-24), 30.5 (C-25), 19.9 (C-26), 20.0 (C-27), 23.5 (C-28), 12.8 (C-29), 103.1 (C-1′), 75.8 (C-2′), 78.9 (C-3′), 72.3 (C-4′), 78.5 (C-5′), 63.4 (C-6′), was identified by comparison to literature data [17]. 

### 2.2. Suppression of Th17-Relevant Cytokines in LPS-Stimulated THP-1 Cells

**Figure 2:**
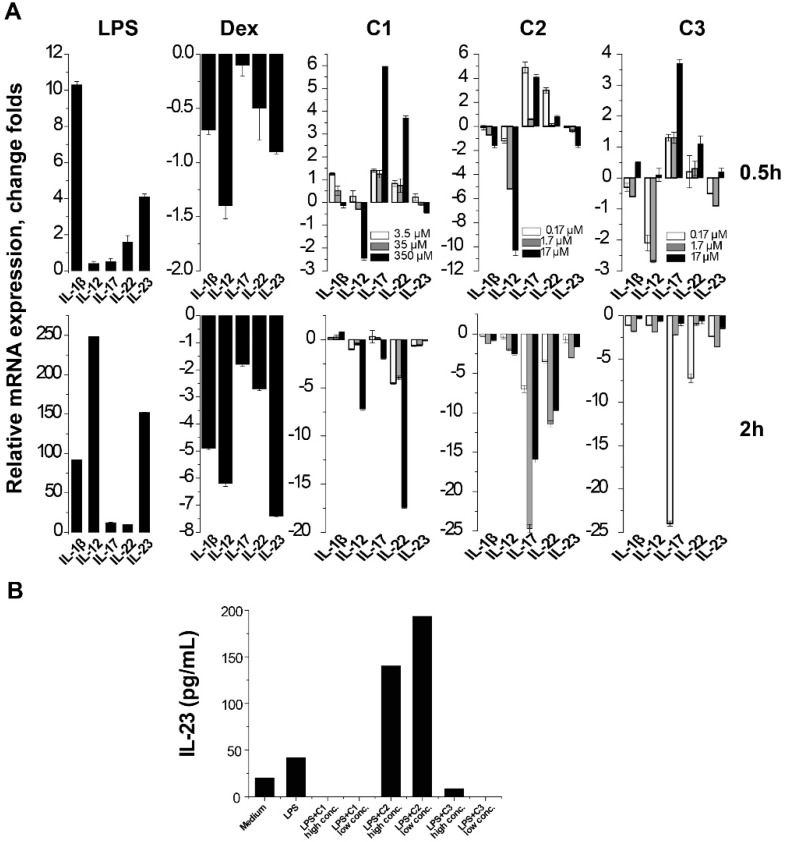
Effects of the three compounds on the expression of pro-inflammatory cytokines in LPS-stimulated THP-1 cells. (**A**) Effects on the cytokine mRNA expressions. Cells were collected at 0.5 h (upper row) or 2 h (lower row) after LPS stimulation. Cells were treated with three concentrations of each compound (indicated in the figure), and 20 nM dexamethasone was used as control. (**B**) The IL-23 levels in the supernatant of overnight cultures were quantified by ELISA. THP-1 cells were treated with C1 (250 and 50 μM), C2 (100 and 20 μM) and C3 (10 and 2 μM), respectively. C1 and C3 in both concentrations decreased the IL-23 remarkably.

Using LPS-stimulated THP-1 cells, we evaluated the effect of the compounds on the expression of a panel of pro-inflammatory cytokines (including IL-1β, IL-8, IL-12 p40, IL-17A, IL-22, IL-23 p19 and TNF-α). As mRNA has a rapid turnover, cells were harvested at both 0.5 h and 2 h for gene expression quantitation. Most cytokines in this panel were upregulated dramatically in a time-dependent manner after LPS-stimulation, and remarkably suppressed by 20 nM dexamethasone (Dex) treatment (Figure 2A). TNF-α and IL-8 expression data were excluded from the figure owing to their poor responses to the three compounds.

Unexpectedly, the three compounds showed a profound inhibitory effect on the expression of Th17-relevant cytokines (IL-17, IL-22 or IL-23). At 0.5 h, all three compounds decreased IL-12/IL-23 p19 mRNA expression. At 2 h, the highest dose of C1 decreased IL-12/IL-23 p19 and IL-22 mRNA expression by 7.2-fold and 17.4-fold, respectively. C2 at 17 μM caused a maximum 24.7-fold reduction in IL-17, and an 11.4-fold reduction in IL-22 mRNA expression, respectively. C3 at 0.17 μM decreased IL-17 by a maximum of 24.0-fold, and IL-22 mRNA expression by 7.2-fold (Figure 2A).

We also determined the levels of IL-23 in the supernatant of THP-1 cells using ELISA kits. After LPS-stimulation, IL-23 levels in the supernatant doubled, but decreased to below the detection limit (16.3 pg/mL) in the presence of either C1 or C3 at both higher and lower concentrations. However, IL-23 levels increased 3- to 4-fold in the presence of C2 (Figure 2B). Levels of IL-17 and IL-22 in the supernatant were too low to detect.

### 2.3. Amelioration of TPA-Induced Psoriasis—Like Skin Lesions

**Figure 3:**
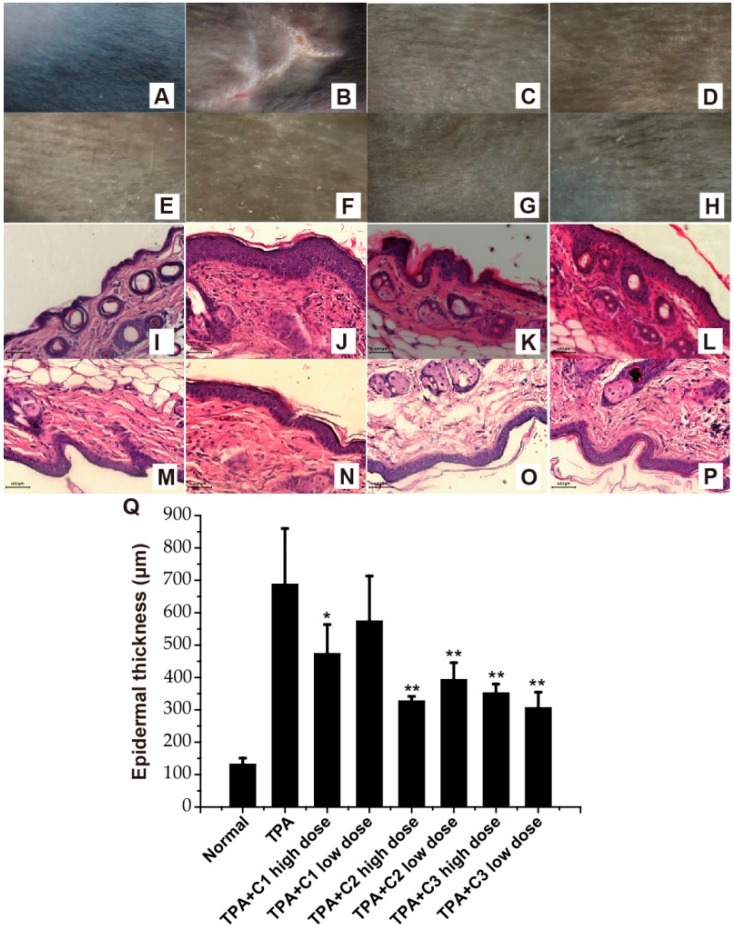
Effects of the three compounds on *12*-*O*-tetradecanoylphorbol-*13*-acetate (TPA)-induced psoriasis-like skin lesions in mice. (**A**–**H**) Shaved dorsal skin of C57BL/6 mice. (**I**–**P**) Pathological changes of dorsal skin of mice, H&E, 40X. Pathological changes emerged in our study included acanthosis and hyperkeratosis, micro-abscess and cellular infiltration. Normal group (**A**,**I**), TPA group (**B**,**J**), and treatment groups (**C**–**H**, and **K**–**P**) treated with high dose or low dose of C1, C2 or C3, respectively. (**Q**) Epidermal thickness of mice (mean ± S.D.). * *p* < 0.05, ** *p* < 0.01 compared with TPA group.

As Th17-relevant cytokines have a critical role in the pathogenesis of psoriasis [14], we evaluated the therapeutic effect of the compounds on TPA-induced psoriasis-like skin lesions in mice. Initially, we compared control and TPA-induced psoriasis-like skin by macroscopic visualization. As reported elsewhere, features commonly found in human psoriatic skin, including erythema and scaling lesions, were not typical in the TPA-treated C57BL/6 mice, but a significant increase in epidermal thickness, with clear evidence of edema, was observed [18]. In our study, macroscopic evaluation indicated that TPA-induced psoriasis-like skin lesions were ameliorated in different degrees in all treated animals after topical administration of the three compounds at both higher and lower concentrations (Figure 3A–H).

Excessive proliferation of epidermal keratinocytes is a typical characteristic of a chronic inflammatory skin condition like psoriasis [19]. For microscopic analysis, standard slide preparation procedures and pathological examinations were performed using dorsal skin samples from the mice. Epidermal thickness was also measured using H&E-stained sections. After topical TPA administration, a thickened epidermis, dilated and congested capillaries, and an increase in infiltrating inflammatory cells could be observed in the local dorsal skin. Topical application of all three compounds ameliorated pathological abnormities significantly, with a noticeable decrease in the epidermal thickness (Figure 3I–P). The protective effects were similar in the C2 and C3 groups, but were less pronounced in the C1 group.

### 2.4. Effect of Compounds on Th17-Relevant Cytokines in Skin

**Figure 4:**
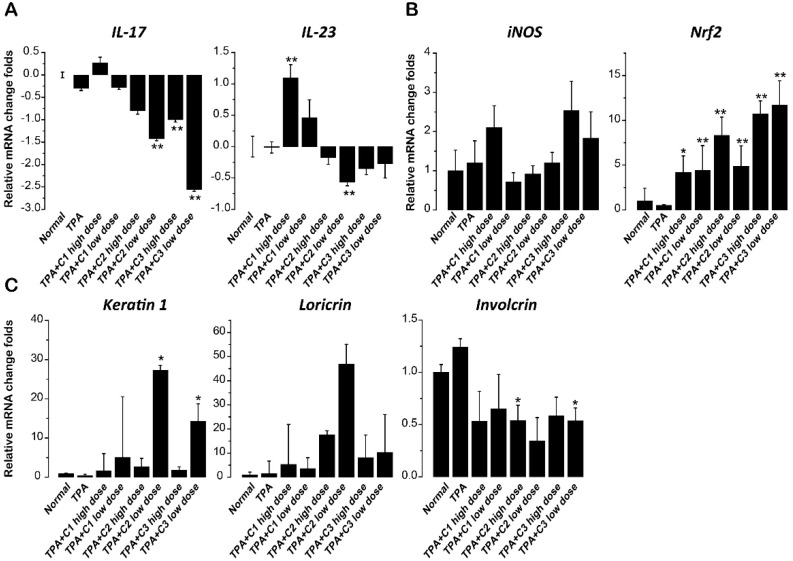
Effects of the three compounds on *12*-*O*-tetradecanoylphorbol-*13*-acetate (TPA)-induced psoriais-like skin lesions in mice. (**A**) Expression changes of IL-17 and IL-23 mRNA. (**B**) Expression changes of iNOS and Nrf-2 mRNA. (**C**) Expression changes of early differentiation marker keratin 1 and late differentiation markers loricrin and involcrin (*geomean* ± *S.E.M.*). * *p* < 0.05, ** *p* < 0.01 compared with TPA group.

We explored whether the three compounds exerted their protective effects by inhibition of Th17-relevant cytokines. Consistent with the microscopically observed relatively weaker protective effect of C1 in comparison to C2 and C3, C1 treatment had no effect on the expression of IL-17A, and even increased the expression of IL-23 in dorsal skin. In contrast, both C2 and C3 treatment resulted in a marked decrease in IL-17A expression and a slight decrease in IL-23 expression in mice, with better protection from TPA-induced skin lesions (Figure 4A).

### 2.5. Effect of Compounds on Inducible NO Synthase (iNOS) and Nuclear Factor Erythroid-2-Related Factor 2 (Nrf2) 

We also investigated the expression of iNOS and Nrf2 in the skin samples, as oxidative stress and cellular redox balance play critical roles in the pathogenesis of psoriasis. As shown in Figure 4B, all the three compounds, especially C2 and C3, increased Nrf2 expression very significantly. They also showed no inhibitory effect on the expression of iNOS.

### 2.6. Effect of Compounds on Differentiation Markers of Keratinocytes

Impaired cornification and terminal differentiation are features of psoriasis. This has been attributed to premature death of the cornifying keratinocytes, which interferes with the expression of late differentiation markers such as profilaggrin and loricrin. These markers are needed to execute full cornification [20]. Treatment with TPA alters the normal differentiation program and suppresses keratin 1 expression, mimicking the incomplete cornification of psoriasis [21]. In our study, C2 and C3 increased the expression of both keratin 1 and loricrin, and decreased the expression of involcrin significantly, consistent with their more profound inhibitory effects on IL-17 and IL-23 expression (Figure 4C).

## 3. Discussion

Psoriasis is one of the most common inflammatory skin diseases and affects more than 2% of the population in Western countries. Aberrant cytokine expression has been proposed as an underlying cause of psoriasis [14]. IL-23, a heterodimeric cytokine composed of p40 (a shared common subunit with IL-12) and p19 subunits, plays a central role in the pathogenesis of psoriasis and was associated with psoriasis in a genome-wide scan [22]. In the IL-23/Th17 axis model of psoriasis, IL-23 is able to produce and induce Th17 cell lymphocyte activation, which subsequently releases IL-17A, IL-17C, IL-17F, IL-22 and other related pro-inflammatory cytokines. Increased expression of these cytokines has been linked to psoriasis [23]. IL-12 p40 [24,25] is a master switch and novel therapeutic target in psoriasis. Anti-IL-12/IL-23p40 antibody ameliorates dermatitis and skin barrier dysfunction in mice with imiquimod-induced psoriasis-like dermatitis [26]. IL-22 is required for imiquimod-induced psoriasis-form skin inflammation in mice [27], and for Th17 cell-mediated pathology [28].

In this study, we isolated three compounds, niazirin, marumoside A and sitosterol-*3*-*O*-*β*-d-glucopyranoside, from *M. oleifera* seeds. All three compounds suppressed the expression of Th17-relevant cytokines (IL-12/IL-23 p40, IL-23 p19, IL-17 and IL-22). Furthermore, we demonstrated that they effectively ameliorated TPA-induced psoriasis-like skin lesions in mice. Our results suggest that the suppression of Th17-relevant cytokines partially explains the anti-inflammatory activities of the compounds. To our knowledge, this is the first report describing the therapeutic application of constituents from *M. oleifera* seeds to psoriasis-like lesions in mice.

Both the iNOS pathway [29] and Nrf2-mediated anti-oxidative defense system [30] are involved in the pathogenesis of psoriasis. Psoriasis vulgaris skin tissues showed increased protein oxidation as well as downregulation of Nrf2, and the activation of Nrf2 might exert therapeutic effects on psoriasis [30]. In addition, the activation of the Nrf2/heme oxygenase-1 (HO-1) pathway enhances STAT3 phosphorylation, which enriched to IL-12b and IL-23a loci and negatively regulated their transcription [31]. Consistent with these reports, we showed the increased Nrf2 expression after treatment with the three compounds, which probably explained their inhibitory effect on IL-23 expression. 

The expression levels of markers of the keratinization process in psoriasis were also used to confirm our results. In psoriasis, the early differentiation marker involucrin is highly expressed, while the other early differentiation factor, keratin 1, and the late differentiation marker, loricrin, are downregulated [20,32]. In our study, we observed a significant decrease in expression of involucrin, as well as increase in expressions of keratin 1 and loricrin, after treatment with the three compounds.

We also noted that the effects of the compounds were not fully dose dependent, and there are discrepancies between different assays in our results, including the discrepancies between the IL-23 concentration in the culture supernatant and the IL-23 mRNA expression in the skin of mice after C1 or C2 treatment. As the turnover of mRNA is rapid, the time-point of sampling could partially explain the discrepancies between results from in vitro and in vivo. In addition, considering the complexity of reciprocal regulations, and that the targets of the three compounds remained unclear, the utilization of different signaling pathways and negative-feedback regulations of IL-23/IL-17 are also possible explanations. Further research in this field to explore the therapeutic targets, mechanisms, and possible clinical applications of compounds from *M. oleifera* would be very useful.

## 4. Materials and Methods

### 4.1. Plant Material

*M. oleifera* seeds were purchased from the Republic of Malawi in accordance with local laws and the guideline of Convention on Biological Diversity, and authenticated by Professor Gang Hao from South China Agricultural University (Guangzhou, Guangdong, China). The voucher specimen (HG-TMI, 5) was deposited at the Institute of Tropical Medicine, Guangzhou University of Chinese Medicine.

### 4.2. Extraction and Isolation

The air-dried and ground *M. oleifera* seeds (2 kg) were extracted three times with EtOH–H_2_O (95:5, *v*/*v*). The combined EtOH extract was evaporated under reduced pressure. After defatting with petroleum ether (60–90 °C), the residue was dissolved in water and extracted successively with EtOAc and *n*-BuOH, respectively. The combined *n*-BuOH mixture (15.7 g) was applied to a D101 macroporous resin (Cangzhou Bao’en Adsorbing Material Technology Co. Ltd., Cangzhou, Hebei, China) column and eluted with MeOH–H_2_O gradient solvents. Combination of similar fractions on the basis of thin-layer chromatography (TLC, Qingdao Haiyang Chemical Co. Ltd., Qingdao, China) analysis afforded seven fractions. Fraction 4 (2.1 g) was applied to a YMC ODS-AQ-HG column (YMC Co. Ltd., Shimogyo-ku, Kyoto, Japan) and eluted with MeOH–H_2_O (10:90, 20:80, 30:70, and 50:50 *v*/*v*) to yield Compound 1 (1.46 g). Fraction 5 (3.7 g) was applied to a reverse-phase silica gel column and eluted with MeOH–H_2_O (20:80, 40:60, and 60:40, *v*/*v*) to yield Compound 2 (1.75 g). The combined EtOAc mixture (885.3 g) was applied to a silica gel (200–300 mesh) column and eluted with CHCl_3_–MeOH gradient solvents. Combination of similar fractions on the basis of TLC analysis afforded three fractions. Fraction 2 (15.9 g) was applied to a silica gel column and eluted with CHCl_3_–MeOH gradients of 95:5, 90:10, and 85:15 (*v*/*v*), respectively, to give Compound 3 (1.32 g). 

### 4.3. General Experimental Procedures for Structural Determination

The ^1^H- and ^13^C-NMR spectra were measured on a Bruker DRX-400 (Bruker Biospin AG, Fällanden, Germany, 400 MHz for ^1^H- and 100 MHz for ^13^C spectra) spectrometer. Chemical shifts were expressed with reference to TMS as the internal standard, and coupling constants (*J*) were given in Hz. HRESIMS was recorded on an Agilent 6210 ESI/TOF mass spectrometer (Agilent Technologies Inc., San Diego, CA, USA).

### 4.4. THP-1 Cell Culture and Compound Treatment

The THP-1 cell line was obtained from the China Center for Type Culture Collection, and grown in RPMI 1640 culture medium (Gibco, Beijing, China) supplemented with fetal bovine serum (Gibco, Paisley, UK), 50 μM β-mercaptoethanol (Sigma, St. Louis, MO, USA), and 10 μg/mL penicillin–streptomycin (Beijing Solarbio Science & Technology penicillin/streptomycin, Beijing, China). Cells were sub-cultured when their density reached 0.7–0.9 million cells/mL.

For experiments, cells were plated in 96-well microplates or 6-well culture plates, and stimulated with 200 ng/mL lipopolysaccharide (LPS, Sigma, St. Louis, MO, USA). The extracted compounds were dissolved in dimethyl sulfoxide (DMSO, Sigma, St. Louis, MO, USA) and diluted with culture medium to obtain the desired concentrations.

### 4.5. RNA Isolation, Reverse Transcription, and Quantitative PCR Analysis

THP-1 cells were lysed directly in the 96-well culture plates using 250 μL of TRIzol reagent (Invitrogen, Carlsbad, CA, USA). After a 5 min incubation, 200 μL of chloroform per 1 mL of TRIzol was added to the tubes for phase separation. After mixing and incubation for 3 min, samples were centrifuged for 15 min at 12,000× *g* at 4 °C, and the aqueous phase was transferred to new tube, mixed with 500 μL isopropanol and 10 μL glycogen, and centrifuged at 12,000× *g* for 15 min at 4 °C. Precipitated RNA pellets were washed with 1 mL of 75% ethanol and centrifuged at 7500× *g* for 5 min at 4 °C. Finally, RNA was dissolved in 10 μL diethyl pyrocarbonate (DEPC)-treated water, and quantified using a NanoDrop 2000 spectrophotometer (Thermo Fisher Scientific Inc., Waltham, MA, USA). RNA was reverse-transcribed using the Super Script III First-Strand Synthesis System for RT–PCR kit (Invitrogen, Carlsbad, CA, USA) according to the manufacturer’s protocol. PCR was performed in duplicate using SYBR Green qPCR master mix (Takara Biomedical Technology Co. Ltd., Dalian, China) in an ABI 7500 Real-Time PCR System (Applied Biosystems, Foster, CA, USA). The cycling program was: denaturation at 95 °C for 15 s, 40 cycles of 95 °C for 15 s and extension at 60 °C for 60 s, followed by melt curve generation. The ΔΔCT method was used to quantify relative mRNA levels as described in User Bulletin 2 (Applied Biosystems). The primer sequences used in this study are available on request.

### 4.6. Enzyme-Linked Immunosorbent Assay (ELISA) Detection of IL-23 in Culture Supernatant

Supernatants of THP-1 cells were collected by centrifugation (for 10 min at 12,000× *g* at 4 °C) 24 h after LPS stimulation, and the levels of IL-23 were determined in duplicate using a human IL-23 Quantikine ELISA Kit (R&D Systems, Minneapolis, MN, USA) according to the manufacturer’s instructions. An ELx808 microplate reader (BioTek, Winooski, VT, USA) was used for absorbance readings and 4-parameter logistic regression was used for data analysis.

### 4.7. Preparation of Ointment

Appropriate amounts of the three compounds were dissolved in 100 μL of DMSO, added to 10 g or 100 g white Vaseline, and thoroughly mixed with an electronic homogenizer (TGrinder OSE-Y10, Beijing, China). Finally, creamy white emulsions, containing 5% or 0.5% (*w*/*w*) C1, 1.5% or 0.15% (*w*/*w*) C2, and 4% or 0.4% (*w*/*w*) C3, respectively, were prepared and stored at 4 °C.

### 4.8. Treatment of TPA-Induced Psoriasis-Like Skin Inflammation in C57BL/6J Mice

Eight-week-old female C57BL/6J mice (20–25 g) were obtained from and housed at the Experimental Animal Center of Guangzhou University of Traditional Chinese Medicine. Studies were performed in accordance with the guidelines approved by the Animal Ethics Committee of Guangzhou University of Traditional Chinese Medicine (approval number: S2017093). All mice were housed in a specific pathogen-free facility with food and water *ad libitum*.

Forty-eight mice were randomly assigned into eight groups, including the control group, model group, and six treatment groups (treated with the high or low dose of C1, C2, or C3, respectively). Animals in the model and treatment groups received 20 μg TPA (Sigma, USA) in 200 μL acetone by topical application to their shaved backs on days 0 and 3, while animals in the control group received only 200 μL acetone.

In the treatment groups, 60 mg of each prepared compound-containing ointment was topically administered to the dorsal skin twice a day for seven consecutive days, while Vaseline-only ointment was similarly administered to the control group as the vehicle control. On days 0 and 3, ointments were given 30 min after TPA treatment. On day 8, all animals were euthanized by cervical dislocation and the TPA/compound-treated dorsal skin samples were harvested. Half of each skin sample was fixed in 10% neutral buffered formalin for histological analysis, while the other half was snap-frozen in liquid nitrogen and stored at 80 °C for mRNA expression studies. The samples were homogenized, and mRNA was extracted and quantified as described previously.

### 4.9. Histology

Fixed skin tissues were embedded in paraffin, cut into 4 μm sections, and stained using hematoxylin and eosin (H&E). Epidermal thickness was measured by an investigator blinded to treatment as described previously [33]. Briefly, maximum epidermal thickness was measured from the tip of the rete ridges to the border of the viable dorsal epidermis using the ocular micrometer of the Leica DMR microscope system (Leica Microsystems, Wetzlar, Germany) at 400×. For each section, the mean value of six measurements was calculated.

### 4.10. Statistics

The statistical significance between groups was assessed by performing one-way ANOVA with the LSD multi-comparison test using R software (Version 3.2.0 for Windows, R Foundation for Statistical Computing) [34].
